# Role of Postoperative Radiotherapy in the Management of Localized Head and Neck Mucosal Melanoma

**DOI:** 10.3390/cancers17081284

**Published:** 2025-04-10

**Authors:** Bong Kyung Bae, Jin Ho Sohn, Dongbin Ahn, Gil Joon Lee, Ji Hye Kwak, Junhee Park, Jeong Eun Lee

**Affiliations:** 1 Department of Radiation Oncology, School of Medicine, Kyungpook National University, Daegu 41944, Republic of Korea; bae8808@gmail.com (B.K.B.); jhp1247@naver.com (J.P.); 2 Department of Otolaryngology-Head and Neck Surgery, School of Medicine, Kyungpook National University, Daegu 41944, Republic of Korea; sohnjh@knu.ac.kr (J.H.S.); godlikeu@naver.com (D.A.); giljoon.lee@gmail.com (G.J.L.); laugh112@naver.com (J.H.K.)

**Keywords:** head and neck mucosal melanoma, postoperative radiotherapy, radiotherapy field, local control, survival

## Abstract

Background/Objectives: To evaluate the role of postoperative radiotherapy (PORT) in the management of localized head and neck mucosal melanoma (HNMM). Methods: We retrospectively reviewed 33 patients who underwent surgery for localized HNMM between 2006 and 2023. PORT was delivered to affected anatomical structures, and elective nodal irradiation was not performed. Treatment outcomes, including local recurrence-free survival (LRFS), progression-free survival (PFS), and overall survival (OS), were analyzed. Results: After surgery, 14 patients (41.4%) received PORT. During follow-up, 87.9% of patients experienced recurrence, with local and distant failures being the most common. PORT significantly improved LRFS (*p* = 0.047) but did not impact PFS or OS. PORT (HR 0.14, *p* = 0.005) and resection margin status (HR 8.71, *p* < 0.001) were significantly associated with LRFS in multivariable analysis. Conclusions: PORT for localized HNMM improved local control. Regional recurrence was rare (one patient, 3.4%) even though ENI was not performed. PORT without ENI could improve clinical outcomes for localized HNMM.

## Simple Summary

The current retrospective study was conducted to evaluate the role of postoperative radiotherapy for localized head and neck mucosal melanoma. Postoperative radiotherapy was delivered to affected anatomical structures without elective nodal irradiation. Postoperative radiotherapy improved local control, and regional recurrence was rare even though elective nodal irradiation was not performed. Postoperative radiotherapy without elective nodal irradiation could improve clinical outcomes for localized head and neck mucosal melanoma.

## 1. Introduction

Head and neck mucosal melanoma (HNMM) is a rare and aggressive malignancy, accounting for approximately 3% of all melanomas and 0.4% to 10% of head and neck melanomas [1,2]. Patients generally exhibit poor prognosis due to the aggressive nature of the tumor, advanced stage at diagnosis, and challenging location of the primary tumor [3,4]. The 5-year overall survival rate ranges from 15% to 25% [3,4].

Surgical resection with an adequate resection margin is generally accepted as the cornerstone of treatment for resectable cases [5]. Despite surgical resection, clinical outcomes remain poor, prompting investigations into the utilization of adjuvant treatment after surgical resection [5,6,7].

Postoperative radiotherapy (PORT) has been shown to improve local control, but its impact on survival remains controversial [8,9,10,11]. The current National Comprehensive Cancer Network guideline strongly recommends PORT with or without systemic therapy as adjuvant treatment [12]. However, the United Kingdom national guideline for HNMM does not recommend routine use of PORT after curative resection [13]. Due to the rarity of the disease, prospective trials focusing specifically on PORT for HNMM are lacking and unlikely to be conducted.

The inconsistency in clinical outcomes regarding PORT may derive from heterogeneity in PORT techniques, such as dose-fractionation schedules and target volume delineation. Despite these technical variations critically influencing treatment outcomes, they are often underreported, potentially explaining the conflicting results in the literature and highlighting the need for studies that provide detailed information on PORT with clinical results [3,14,15].

To address these points, we have conducted this retrospective study with the following objectives. First, to report clinical outcomes of patients with localized HNMM after surgery with curative intent. Second, acknowledging that there are no or very limited published studies focusing on the details of PORT, we aimed to report the details of the PORT field and dose utilized in our institution and investigate the role and impact of PORT. Third, we aim to report the pattern of recurrence by reporting the sites of recurrence, with the goal of using these findings to help determine more appropriate and effective future treatment strategies for this rare and aggressive malignancy.

## 2. Materials and Methods

### 2.1. Study Design

This retrospective study was approved by the Institutional Review Board of the Kyungpook National University Hospital (KNUH 2024-03-031) and the Kyungpook National University Chilgok Hospital (KNUCH 2024-03-043). Informed consent was waived due to the retrospective nature of this study.

We reviewed medical records of patients who underwent surgery for HNMM between 2006 and 2023 in our institutions. Patients who underwent palliative surgery, patients with regional or distant metastasis, or patients who were lost to follow-up within 6 months after surgery were excluded. Patients who underwent salvage surgery for local recurrence were classified as new, distinct cases and included in this study. In total, 33 patients (26 initial surgeries and 7 salvage surgeries) who underwent curative intent surgery for localized HNMM with a median follow-up of 2.2 years (range, 0.2–11.5 years) were included in this study.

### 2.2. Postoperative Radiotherapy

PORT was primarily recommended for patients with T4 disease or resection margin concerns. It was also recommended that the primary surgeon consider whether PORT was necessary for the patient. The PORT policies and dose schedules of our institutions were as follows. PORT was delivered 4 to 8 weeks after surgery with intensity-modulated radiotherapy with a simultaneous integrated boost technique. Planning CT was acquired with a slice thickness of 2.5 to 3 mm. The patient was positioned supine and immobilized using a thermoplastic mask. The tumor bed was defined as the high-risk clinical target volume (CTV). Affected anatomical structures, such as the nasal cavity and left maxillary sinus for cancers originating from the left nasal cavity, were defined as low-risk CTV. The elective nodal area was not included in the CTV. Planning target volumes (PTVs) were generated by expanding 3 mm in all directions from CTVs. A total of 66 Gy in 30 fractions (2.2 Gy per fraction) was prescribed for the high-risk PTV, and 48 Gy in 30 fractions (1.6 Gy per fraction) was prescribed for the low-risk PTV. Adjustments from the PORT policies were made at the primary physician’s discretion.

### 2.3. Assessments

We retrospectively collected baseline clinical and pathological information, including age, sex, tumor location, stage, resection margin status, and treatment details from the medical records. All patients underwent neck computed tomography for locoregional staging and positron emission tomography-computed tomography for distant staging. Patients were re-staged according to the American Joint Committee on Cancer 8th edition staging manual [16]. Recurrences were categorized into three groups based on the location of the recurrent lesion. For sinonasal origin cancers, local recurrence was defined as recurrence within the nasal cavity or paranasal sinuses (PNS). For oral cavity origin cancers, local recurrence was defined as recurrence in the oral cavity or oropharynx. Regional recurrence was defined as recurrence in the neck lymphatic chain, and distant metastasis was defined as recurrence outside the local and regional areas. Event-free survivals were defined as the time interval between surgery and the occurrence of corresponding events (local, regional, or distant failure, or death). Loss to follow-up or death without the specific events were treated as censored events for the respective survivals.

### 2.4. Statistical Analysis

Variables are presented as medians with ranges or frequencies with percentages, as appropriate. The Chi-square test or Fisher’s exact test was performed to compare categorical variables, and a *t*-test was performed to compare continuous variables between groups. Survival data were analyzed using the Kaplan–Meier method and compared using the log-rank test. Cox regression analysis was performed to determine the risk factors of local recurrence-free survival (LRFS), progression-free survival (PFS), and overall survival (OS). Variables with a *p*-value of < 0.10 in univariable analysis were included in the multivariable analysis. The final multivariable model was determined using a backward variable selection method with an elimination criterion of 0.05. A *p*-value of <0.05 was considered to indicate statistical significance. Statistical analyses were performed using IBM SPSS Statistics for Windows (version 27.0; IBM Corp., Armonk, NY, USA).

## 3. Results

### 3.1. Patients

The median age of the patients at the time of surgery was 76 years (range, 40–86 years). The majority of patients were male (22 patients, 66.7%), had sinonasal origin (27 patients, 81.9%), and had T3 stage disease (25 patients, 75.8%). All patients underwent surgical excision of the primary tumor. The resection margin status was clear in 22 patients (66.7%) and positive in 11 patients (33.3%). After surgery, 14 patients (41.4%) received PORT with a median total dose of 66 Gy (range, 59.4–70 Gy) with conventional fractionation. All patients who received PORT completed the planned treatment. The proportion of T4 patients was significantly higher for the patients who received PORT (six patients, 42.9%) compared to surgery alone (two patients, 10.5%) (*p* = 0.047). Although statistically insignificant, the proportion of salvage treatment was higher in surgery alone (six patients, 31.6%) than in PORT (one patient, 7.1%). The majority of prior treatments before salvage treatment were surgery alone (six patients). There were no significant differences observed in other baseline variables. Table 1 summarizes the baseline clinical characteristics of the patients.

### 3.2. Failure Pattern

**Figure 1:**
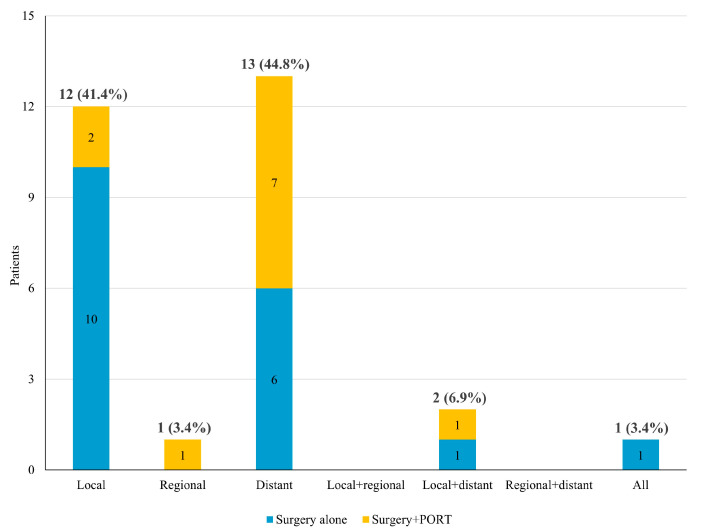
Stacked column chart showing the pattern of recurrence after surgery alone (blue) and PORT after surgery (yellow). PORT, postoperative radiotherapy.

During follow-up, 29 patients (87.9%) experienced recurrence after surgery. Median time to recurrence was 0.9 years (range, 0.1–3.0 years), with more than half of the recurrences occurring within one year after surgery (18 patients, 62.0%). The initial recurrence sites were local in 12 patients (41.4%), regional in one patient (3.4%), distant in 13 patients (44.8%), local and distant in two patients (6.9%), and local, regional, and distant in one patient (3.4%). Although the number of patients who experienced local recurrence was higher in the surgery alone group (10 patients) compared to the PORT group (two patients), the difference was not significant (*p* = 0.166) (Figure 1).

Table 2 summarizes the characteristics of the respective local failures. For sinonasal origin cancers, the recurrence sites were the nasal cavity in 9 patients, the ethmoidal sinus in three patients, and the maxillary sinus in one patient. For two oral cavity origin cancers, recurrence occurred in oropharynx. The local recurrence sites of patients who underwent PORT were within the RT field: nasal cavity (two patients, within high-risk CTV) and ethmoidal sinus (one patient, within low-risk CTV).

Regional recurrence occurred in one patient. The patient experienced left preauricular, intra-parotid, level 1b lymph node recurrence 5 months after surgery and PORT for HNMM in the left nasal cavity. Regional relapse free survival was 96.8% with 95% confidence interval of 90.5 to 100%.

### 3.3. Survival and Risk Factors

**Figure 2:**
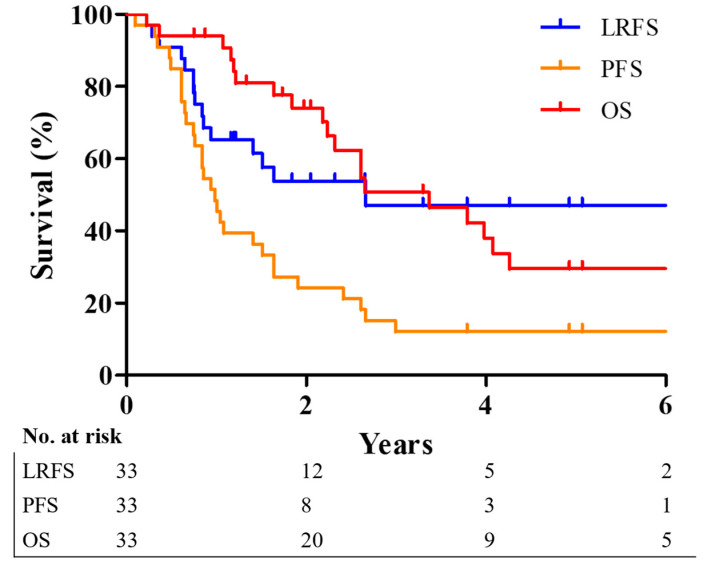
Kaplan–Meier survival curves showing the LRFS (blue), PFS (orange), and OS (red) of patients. LRFS, local recurrence-free survival; PFS, progression-free survival; OS, overall survival.

The 2- and 5-year survival rates were 53.8% and 47.1% for LRFS, 21.2% and 12.1% for PFS, and 74.1% and 29.6% for OS, respectively. The Kaplan–Meier survival curves for the respective survival rates are illustrated in Figure 2.

Cox regression analyses were performed to identify risk factors of respective survivals. For LRFS, resection margin status (hazard ratio [HR], 4.20; 95% CI, 1.50–11.75; *p* = 0.006) was a statistically significant risk factor in univariable analysis. In the multivariable analysis, PORT after surgery (HR, 0.14; 95% CI, 0.04–0.55; *p* = 0.005) and resection margin status (HR, 8.71; 95% CI, 2.71–28.02; *p* < 0.001) were independently associated with LRFS. For PFS, ages older than 70 years were significantly associated with worse survival in both univariable (HR, 2.39; 95% CI, 1.09–5.24; *p* = 0.029) and multivariable (HR, 2.39; 95% CI, 1.09–5.24; *p* = 0.029) analyses. Age (HR, 4.47; 95% CI, 1.48–13.46; *p* = 0.008), T stage (HR, 4.03; 95% CI, 1.48–10.96; *p* = 0.006), and resection margin status (HR, 3.05; 95% CI, 1.18–7.86; *p* = 0.021) were significant risk factors for OS in univariable analysis. In multivariable analysis, age (HR, 3.63; 95% CI, 1.18–11.23; *p* = 0.024) and T stage (HR, 3.05; 95% CI, 1.11–8.41; *p* = 0.031) were found to be independent risk factors for OS. The results of the Cox regression analyses are summarized in Table 3 (LRFS), and Supplementary Tables S1 (PFS) and S2 (OS).

**Figure 3:**
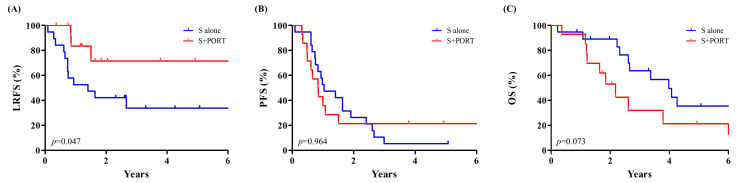
Comparison of LRFS (**A**), PFS (**B**), and OS (**C**) between patients who underwent surgery alone (blue) and PORT after surgery (red). LRFS, Local recurrence-free survival; PFS, progression-free survival; OS, overall survival; PORT, postoperative radiotherapy; S, surgery.

Figure 3 shows Kaplan–Meier survival curves for LRFS (A), PFS (B), and OS (C) stratified by treatment arm: surgery alone versus PORT after surgery. Though the proportion of T4 patients was significantly higher in the PORT arm (*p* = 0.047, Table 1), PORT after surgery significantly improved LRFS compared to surgery alone (2-year, 71.4% vs. 42.1%, *p* = 0.047). However, PORT did not significantly improve PFS (2-year, 21.4% vs. 21.1%, *p* = 0.964) or OS (2-year, 53.1% vs. 82.8%, *p* = 0.073).

### 3.4. Toxicity Profiles of PORT

Following PORT, grade 2 acute toxicity was observed in four patients (28.6%). These events consisted of radiation mucositis (three patients) and radiation dermatitis (one patient), which were all safely managed without significant complications. No grade 3 or higher acute toxicities were recorded, and no late toxicities were observed.

## 4. Discussion

In this study, we retrospectively reviewed the clinical outcomes of patients who underwent surgery with curative intent for localized HNMM. Overall, the clinical outcomes were poor, with a 2-year LRFS of 53.8%, PFS of 21.2%, and OS of 74.1%. More than 80% of patients experienced recurrence after surgery. Most of the recurrences were local or distant, and regional recurrence was rare even after PORT without ENI. PORT was a significant factor in LRFS. To the best of our knowledge, this study appears to be the first to report specific details regarding both PORT field delineation and prescribed doses for HNMM. We believe that presenting this detailed PORT methodology with the clinical outcomes provides information of significant value for clinical practice. Although the sample size of the current study was small, considering the rarity of HNMM, our findings add value to the existing literature and clinical understanding.

Surgical resection with a clear resection margin is considered the mainstay treatment for resectable HNMM [5,9,17,18]. However, even after surgical resection, many patients experience disease failure, with reported 5-year survival rates ranging from 40% to 79% for LRFS (this study, 47.1%) and from 4% to 40% for PFS (this study, 12.1%) [18,19,20,21]. Additional treatments beyond surgery should be considered to improve patient prognosis. However, there are no clinical trials or definite guidelines to guide treatment decisions, and the efficacy of possible adjuvant treatment options has not been clarified [22,23,24].

Evaluating the pattern of failure is crucial for determining appropriate treatment strategies. Amit et al. reported that for sinonasal mucosal melanoma patients, distant metastasis was the most common cause of treatment failure (35%), followed by local (18%) and regional (11%) recurrence [8]. Xu et al. reported that in their analysis of 262 stage III-IVb HNMM patients, distant metastasis was observed in 40.8% of patients, followed by local (28.2%) and regional (19.8%) recurrence [24]. Lee et al. retrospectively reviewed 31 patients who underwent surgery-based treatment for HNMM [9]. They reported that the most common recurrence site was distant (23%) followed by local (19%), multiple (16%), and regional (3%) sites. Consistent with these studies, the most common recurrence sites in our patient cohort were local and distant, with regional recurrence being minimal (Figure 1).

The role of PORT in the management of HNMM has been reported in multiple studies. Temam et al. retrospectively reviewed 69 HNMM patients who underwent surgery for definitive treatment. They reported that PORT was associated with improved local disease-free survival (median local disease-free survival, 9 months vs. 33 months) [25]. Yao et al. conducted a single-arm phase II clinical trial to evaluate the effectiveness and safety of primary surgery with PORT for treating HNMM [26]. They reported that PORT resulted in excellent local control (3-year LRFS, 91.7%), but high distant metastasis rates limited survival for this group of patients. Benlyazid et al. retrospectively reviewed 160 non-metastatic HNMM patients treated with surgery alone or with PORT at French medical institutions [1]. They reported that PORT improved locoregional disease control (5-year cumulative incidence of locoregional recurrence, 55.6% vs. 29.9%). However, PORT patients had advanced disease, and a higher rate of distant metastasis was observed in the group. Grant-Freemantle et al. performed a systematic review and meta-analysis to assess the effectiveness of RT in the treatment of HNMM [3]. They concluded that PORT showed a moderate survival advantage compared to surgery, likely due to reduced local recurrence. The pooled risk ratio for local recurrence at 5 years post treatment was 0.63 (*p* = 0.005) in favor of PORT. Like these previously reported studies, our study showed that PORT results in improved LRFS (2-year, 71.4% vs. 42.1%, *p* = 0.047); however, this improvement did not translate into PFS or OS gain (Figure 3).

There could be several reasons why the improved local control in PORT did not translate into PFS or OS gain. Primarily, the aggressive nature and high propensity for distant metastasis of HNMM could have affected the survival outcomes irrespective of local control [11,27]. Additionally, the retrospective nature of the current study resulted in differences in baseline patient characteristics between the treatment groups and could have affected the clinical outcome comparisons. A significantly higher proportion of T4 patients received PORT (Table 1). Despite that, LRFS was significantly better for the PORT group, and OS was worse for the PORT group, though statistically insignificant (*p* = 0.073, Figure 3).

Recently, two large multicenter retrospective studies have focused on the clinical outcomes of HNMM. Lechner et al. conducted a retrospective analysis of 505 sinonasal melanoma cases [14]. Their findings indicated that PORT significantly improved OS (HR, 0.74; *p* = 0.021) and showed a trend toward better LRFS (HR, 0.62; *p* = 0.066) compared to surgery alone. Additionally, using immune checkpoint inhibitors for recurrent or persistent cases resulted in a significant OS benefit (HR, 0.50; *p* = 0.036). In contrast, a retrospective multicenter study by Scheurleer et al., which included 320 sinonasal mucosal melanoma patients, reported conflicting results [15]. They reported that PORT did not improve outcomes compared to surgery alone (HR, 1.16; 95% CI, 0.80–1.68). They did not explore the role of immunotherapy, as only eight patients received such treatments. The reason for the differing findings regarding PORT’s role between the two studies remains unclear. Since both studies are large-scale multicenter cohort studies, variations the practice patterns and treatment aims across centers may have contributed to the inconsistencies. Neither study provided details about the specific details of the PORT, such as the aim of the PORT, RT field, or RT dose. These factors may have influenced clinical outcomes, making it difficult to draw definitive conclusions.

While published studies have reported that PORT results in improved LRFS, the PORT dose is heterogeneous within and between studies, and the RT target volume is not clearly defined [1,14,15,25,26]. In this study, we performed homogeneous PORT, 66 Gy in 30 fractions to the high-risk tumor bed and 48 Gy in 30 fractions to affected anatomical structures with no elective nodal irradiation (ENI). Although the number of patients was small, our evaluation provides valuable information for PORT target delineation. We believe that the following regions should be included in the PORT field for HNMM: for the sinonasal region: nasal cavity, ethmoidal sinus, and ipsilateral maxillary sinus; for the oral cavity region: oral cavity and oropharynx. As regional recurrence was limited, ENI did not seem necessary.

With the introduction of immunotherapy, significant improvements have been achieved in the management of cutaneous melanoma [6]. However, it is challenging to draw conclusions for mucosal melanoma due to the rarity of the disease. Several post-hoc analyses of mucosal melanoma patients treated with immunotherapy have reported improved clinical outcomes [28,29]. However, these results should be interpreted with caution due to the post hoc nature of the studies, differences in baseline clinical characteristics, and the small sample size. Based on the positive results, the guidelines suggested by the United Kingdom HNMM guideline development group recommended the use of immunotherapy and BRAF-targeted therapies as adjuvant treatments [13]. In this study, none of the patients received adjuvant immunotherapy after surgery. The incorporation of immunotherapy in the management of mucosal melanoma seems promising. Improving local disease control with PORT and distant disease control with immunotherapy could potentially lead to improved survival. Further studies are warranted to assess the effectiveness of the adjuvant treatments.

In this study, we reported the clinical outcomes of patients with localized HNMM who underwent surgery with curative intent. Notably, we reported local recurrence sites for patients with local failure. We believe that this study has shown the role of PORT for localized HNMM and provides valuable information for PORT field delineation.

This study has several limitations. First, due to the retrospective nature of the study and the small sample size, important clinical factors may have been masked or overestimated. However, it is of note that the disease is very rare, and this study focused on a very specific group of patients within this disease entity, providing useful information for the treatment of localized HNMM. Second, we included patients who not only underwent initial surgery but also underwent salvage surgery for local recurrence. We included the latter group because, although the patients were recurrent cases, the recurrences were minimal and were considered resectable with curative intent surgery for local recurrences. However, recurrent patients may exhibit more aggressive features compared to those initially diagnosed. Although the difference in the number of recurrent cases between treatment groups was not statistically significant, it should be noted (Table 1). Therefore, the outcomes of this study should be interpreted with caution and accepted more conservatively.

## 5. Conclusions

In conclusion, PORT after surgery for localized HNMM resulted in improved local control. Compared with local and distant metastasis, regional recurrence was rare. The local recurrence sites were the nasal cavity, ethmoidal sinus, and maxillary sinus for sinonasal origin cancers, and oropharynx for oral cavity origin cancers. Including these sites in the PORT field should be considered, while ENI does not seem to be necessary. Future studies incorporating immunotherapy with PORT after surgery for HNMM are warranted to further improve clinical outcomes.

## Figures and Tables

**Table 1:** Baseline clinical characteristics.

Variables	All Patients	Surgery Alone	Surgery + PORT	*p*-Value
	n = 33	n = 19	n = 14	
Age (Years)				0.337
Median, range	76 (40–86)	76 (51–81)	71 (40–86)	
Sex				0.803
Male	22 (66.7%)	13 (68.4%)	9 (64.3%)	
Female	11 (33.3%)	6 (31.6%)	5 (35.7%)	
Primary site				1.000
Sinonasal	27 (81.8%)	16 (84.2%)	11 (78.6%)	
Other	6 (18.2%)	3 (15.8%)	3 (21.4%)	
T stage				0.047
T3	25 (75.8%)	17 (89.5%)	8 (57.1%)	
T4	8 (24.2%)	2 (10.5%)	6 (42.9%)	
N stage				1.000
N0	33 (100.0%)	19 (100.0%)	14 (100.0%)	
N1	0 (0.0%)	0 (0.0%)	0 (0.0%)	
Resection margin				0.803
Clear	22 (66.7%)	13 (68.4%)	9 (64.3%)	
Positive	11 (33.3%)	6 (31.6%)	5 (35.7%)	
Treatment				0.090
Initial	26 (78.8%)	13 (68.4%)	13 (92.9%)	
Salvage	7 (21.2%)	6 (31.6%)	1 (7.1%)	
Prior treatment				1.000
Surgery alone	6 (85.7%)	5 (83.3%)	1 (100.0%)	
Surgery + PORT	1 (14.3%)	1 (16.7%)	0 (0.0%)	

PORT, postoperative radiotherapy.

**Table 2:** Local recurrence sites of the patients.

Patient	Origin	T Stage	RM	PORT	LRFS	Recur Site	RT Field
1	Maxillary sinus	4	Positive	Yes	1.5	Nasal cavity	High-risk CTV
2	Nasal cavity	3	Positive	Yes	0.9	Nasal cavity	High-risk CTV
3	Nasal cavity	3	Clear	Yes	0.8	Ethmoidal sinus	Low-risk CTV
4	Nasal cavity	4	Positive	No	0.1	Nasal cavity	-
5	Nasal cavity	3	Positive	No	0.3	Nasal cavity	-
6	Nasal cavity	3	Positive	No	0.7	Nasal cavity	-
7	Nasal cavity	3	Positive	No	1.4	Nasal cavity	-
8	Nasal cavity	3	Positive	No	0.3	Ethmoidal sinus	-
9	Nasal cavity	3	Positive	No	0.6	Maxillary sinus	-
10	Nasal cavity	4	Clear	No	0.7	Ethmoidal sinus	-
11	Nasal cavity	3	Clear	No	0.8	Nasal cavity	-
12	Nasal cavity	3	Clear	No	0.9	Nasal cavity	-
13	Nasal cavity	3	Clear	No	2.7	Nasal cavity	-
14	Oral cavity	3	Clear	No	0.7	Oropharynx	-
15	Oral cavity	3	Clear	No	1.6	Oropharynx	-

RM, Resection margin; PORT, postoperative radiotherapy; LRFS, local recurrence-free survival; CTV, clinical target volume.

**Table 3:** Results of Cox regression analyses for LRFS.

Variable		Univariable		Multivariable	
		HR (95% CI)	*p* Value	HR (95% CI)	*p* Value
Age			0.333		
	<70 years	1.00			
	≥70 years	1.68 (0.59–4.77)			
Sex			0.601		
	Male	1.00			
	Female	1.36 (0.43–4.27)			
Primary site			0.402		
	Sinonasal	1.00			
	Other	1.72 (0.48–6.11)			
T stage			0.962		
	T3	1.00			
	T4	1.03 (0.29–3.67)			
Disease status			0.157		
	Initial	1.00			
	Recurrent	2.31 (0.73–7.33)			
Treatment			0.061		0.005
	Surgery	1.00		1.00	
	Surgery + PORT	0.30 (0.08–1.06)		0.14 (0.04–0.55)	
RM			0.006		<0.001
	Clear	1.00		1.00	
	Positive	4.20 (1.50–11.75)		8.71 (2.71–28.02)	

LRFS, Local recurrence-free survival; RM, resection margin; PORT, postoperative radiotherapy.

## Data Availability

Data that support the findings of this study are available from the corresponding author upon reasonable request.

## Supplementary Materials

The following supporting information can be downloaded at https://www.mdpi.com/article/10.3390/cancers17081284/s1, Supplementary Table S1. Results of Cox regression analyses for PFS; Supplementary Table S2. Results of Cox regression analyses for OS.
