# Supplementary material for: Role of Postoperative Radiotherapy in the Management of Localized Head and Neck Mucosal Melanoma
Source: Cancers (Basel). 2025 Apr 10;17(8):1284. doi: 10.3390/cancers17081284 (PMC12025539; doi:10.3390/cancers17081284)
Supplement: Supplementary file 1 [file cancers-17-01284-s001.zip › cancers-3542550-supplementary.pdf]

Supplementary Table S1. Results of Cox regression analyses for PFS

**Variable**		**Univariable HR (95% CI)**	**p value**	**Multivariable HR (95% CI)**	**p value**
Age			0.029		0.029
	< 70 years	1.00		1.00	
	≥ 70 years	2.39 (1.09-5.24)		2.39 (1.09-5.24)	
Sex			0.971		
	Male	1.00			
	Female	0.99 (0.46-2.13)			
Primary site			0.090		
	Sinonasal	1.00			
	Other	0.47 (0.18-1.13)			
T stage			0.153		
	T3	1.00			
	T4	1.87 (0.79-4.41)			
Disease status			0.290		
	Initial	1.00			
	Recurrent	1.60 (0.67-3.79)			
Treatment			0.964		
	Surgery	1.00			
	Surgery + PORT	0.98 (0.46-2.09)			
RM			0.190		
	Clear	1.00			
	Positive	1.69 (0.77-3.68)			

PFS, Progression-free survival; RM, Resection margin; PORT, Postoperative radiotherapy

Supplementary Table S2. Results of Cox regression analyses for OS

**Variable**		**Univariable HR (95% CI)**	**p value**	**Multivariable HR (95% CI)**	**p value**
Age			0.008		0.024
	< 70 years	1.00		1.00	
	≥ 70 years	4.47 (1.48-13.46)		3.63 (1.18-11.23)	
Sex			0.099		
	Male	1.00			
	Female	0.47 (0.19-1.15)			
Primary site			0.972		
	Sinonasal	1.00			
	Other	1.02 (0.40-2.58)			
T stage			0.006		0.031
	T3	1.00		1.00	
	T4	4.03 (1.48-10.96)		3.05 (1.11-8.41)	
Disease status			0.610		
	Initial	1.00			
	Recurrent	0.73 (0.21-2.48)			
Treatment			0.059		
	Surgery	1.00			
	Surgery + PORT	2.37 (0.97-5.78)			
RM			0.021		
	Clear	1.00			
	Positive	3.05 (1.18-7.86)			

OS, Overall survival; RM, Resection margin; PORT, Postoperative radiotherapy
